# A survey of foot orthoses prescription habits amongst podiatrists in the UK, Australia and New Zealand

**DOI:** 10.1186/s13047-018-0304-z

**Published:** 2018-11-26

**Authors:** Lara S. Chapman, Anthony C. Redmond, Karl B. Landorf, Keith Rome, Anne-Maree Keenan, Robin Waxman, Begonya Alcacer-Pitarch, Heidi J. Siddle, Michael R. Backhouse

**Affiliations:** 1 Department of Podiatry, Harrogate and District NHS Foundation Trust, Harrogate District Hospital, Lancaster Park Road, Harrogate, UK; 2 Leeds Institute of Rheumatic and Musculoskeletal Medicine, University of Leeds, Leeds, UK; 3 NIHR Leeds Biomedical Research Centre, Leeds Teaching Hospitals NHS Trust, Leeds, UK; 4 Discipline of Podiatry, School of Allied Health, La Trobe University, Melbourne, Australia; 5 La Trobe Sport and Exercise Medicine Research Centre, School of Allied Health, La Trobe University, Melbourne, Australia; 6 Health and Rehabilitation Research Institute and School of Podiatry, Auckland University of Technology, Auckland, New Zealand; 7 School of Healthcare, University of Leeds, Leeds, UK; 8 York Trials Unit, Department of Health Sciences, University of York, York, UK

**Keywords:** Foot, Orthoses, Podiatry, Survey

## Abstract

**Background:**

Foot orthoses are frequently used but little is known about which types are used in contemporary practice. This study aimed to explore the types of foot orthoses currently used by podiatrists and the prescription variations in a range of conditions.

**Methods:**

A web-based, cross-sectional survey was distributed through professional bodies in the United Kingdom (UK), Australia, and New Zealand. Questions focussed on foot orthosis prescription habits in relation to 26 conditions affecting the back and lower limb.

**Results:**

Two hundred and sixty-four podiatrists practising in 19 different countries completed the survey; the majority practised in the UK (47%, *n* = 124), Australia (30%, *n* = 79) and New Zealand (12%, *n* = 32). Respondents qualified between 1968 and 2016, and 147 (56%) were female. Respondents worked in different healthcare sectors and this varied between countries: 42 (34%) respondents in the UK worked solely in the public sector, compared to 3 (4%) in Australia and 2 (6%) in New Zealand. Forty-four (35%) respondents in the UK worked solely in private practice, compared to 64 (81%) in Australia and 14 (44%) in New Zealand.

UK respondents prescribed more prefabricated orthoses per week (mean 5.5 pairs) than simple insole-type devices (±2.7) and customised devices (±2.9). Similarly, respondents in New Zealand prescribed more prefabricated orthoses per week (±7.7) than simple (±1.4) and customised (±2.8) devices. In contrast, those in Australia prescribed more customised orthoses per week (±4.4) than simple (±0.8) and prefabricated (±1.9) orthoses. Differences in the types of orthoses prescribed were observed between country of practice, working sector, and the condition targeted. Generally, prefabricated orthoses were commonly prescribed for the 26 highlighted conditions in the UK and New Zealand. Australian podiatrists prescribed far fewer devices overall, but when they did prescribe, they were more likely to prescribe custom devices. Respondents in all three countries were more likely to prescribe customised orthoses for people with diabetes complicated by peripheral neuropathy than for diabetes without this complication.

**Conclusions:**

Foot orthosis prescription habits vary between countries. Prefabricated orthoses were frequently prescribed in the UK and New Zealand, and customised orthoses in Australia. Prescriptions for people with diabetes differed depending on the presence of neuropathy, despite a lack of robust evidence supporting these decisions. This study provides new insight into contemporary practice.

**Electronic supplementary material:**

The online version of this article (10.1186/s13047-018-0304-z) contains supplementary material, which is available to authorized users.

## Background

Foot orthoses (FOs) are frequently prescribed by podiatrists for a variety of foot and lower limb conditions. They range considerably in terms of their design but can be broadly divided into two categories: (i) accommodative or simple FOs, which provide cushioning and offloading of structures, or (ii) functional FOs, which aim to systematically alter abnormal foot mechanics and function to alleviate symptoms. Functional FOs can be further categorised as either customised or prefabricated FOs [1]. A ‘customised’ FO is tailored to the individual based on a 3-dimensional (3D) model of the plantar aspect of the foot, whereas prefabricated FOs are mass-produced to a generic foot shape [2, 3].

Although their exact mechanism of action remains unclear, FOs are thought to alter the mechanics of the feet and lower limb in a systematic way. Both accommodative and functional FOs are reported to help a range of lower limb pathologies [4–9] by alleviating symptoms [8, 9] improving function [10], preventing deformity [11], and preventing injury [12]. As such, they are now recommended in national guidelines for a number of conditions, including rheumatoid arthritis [13], osteoarthritis (OA) [14], and diabetes [15].

The cost of providing FOs remains an ongoing issue for healthcare providers, patients and practitioners. A recent exploratory clinical trial [16] that also evaluated cost effectiveness found that a pair of customised FOs were £52.60 more expensive to produce than a simple FO when costed from a National Health Service (NHS) perspective, and £80.00 more expensive from an NHS and societal perspective. The authors of this trial attributed these additional costs to manufacturing time and cost of materials used in the manufacturing process. Similarly, the labour, materials and laboratory costs incurred in a previous trial [3] indicated that customised FOs were 3.5 times more costly than prefabricated devices at the point of issue. Clearly, cost differences exist between these orthotic devices, however there is evidence that indicates similarities in function between certain FO types. For example, studies have shown that some contoured prefabricated FOs exhibit similar physical characteristics to customised FOs [3, 17], and can reduce patient symptoms for certain conditions to the same extent as customised devices [5]. The use of prefabricated FOs may therefore lead to substantial potential healthcare cost savings without compromising treatment effectiveness [18].

Although the use of FOs in clinical practice appears to be widespread, evidence and guidelines relating to the prescription of FOs are limited, and consequently there is no broad consensus on the best type of insole to use for specific conditions. FO prescriptions may therefore vary extensively between clinicians [19, 20], but very little is known about how prescription habits differ across countries and according to pathology. In 2001, Landorf et al. [7] explored the types of FOs used within podiatric practice in Australia and New Zealand and found that the majority (72%) of respondents prescribed customised FOs most often, although respondents in New Zealand were three times more likely than those in Australia to prescribe prefabricated FOs. More recently, Menz et al. [21] analysed prescription characteristics of custom-made foot orthoses from a single orthotic laboratory in Australia, and identified that prescriptions were influenced by the extent of the patient’s rearfoot pronation, the age and sex of the patient, and the clinician’s geographic location. Nester et al. [22] surveyed podiatrists, physiotherapists and orthotists in the United Kingdom (UK), and found that prefabricated FOs were used by 93% of respondents, although 75% also provided customised FOs as part of their practice.

Despite comparable education and scope of podiatric practice, no previous studies have directly compared FO prescription habits among podiatrists in the UK, Australia and New Zealand. Furthermore, no research has to date provided insight into the types of FOs prescribed for specific presentations or conditions commonly affecting the lower limb. Information on the FOs currently in use is vital to establish a benchmark of contemporary practice, ensuring that future research evaluating the clinical and cost effectiveness of FOs is relevant to practice.

The aims of the current study were to describe and compare the types of FOs currently prescribed by podiatrists across the UK, Australia and New Zealand, and to determine whether the type of FO prescribed varies between a range of common presentations and conditions affecting the back and lower limb.

## Methods

The study utilised a cross-sectional, online, self-administered survey to elicit the FO prescription habits of registered podiatrists in the UK, Australia and New Zealand. Ethical approval was received from the School of Medicine Research Ethics Committee, University of Leeds (Ref: MREC15–052). Subsequent approval was also gained from La Trobe University (Ref: MREC15–052) and Auckland University (Ref: 16/133) of Technology. Consent was implied by completion of the survey and it was accessible from June 2016 to November 2016.

### Survey design

Survey questions were developed by experienced clinicians and experts in FO research and piloted with user groups of local podiatrists. The final survey (Additional file 1) consisted of 29 questions that were arranged into three sections. The first section of the survey was designed to elicit demographic and descriptive data from participants, including gender, year qualified, country of practice, country in which primary podiatry qualification was undertaken, percentage of clinical time spent working in the public sector and private practice, and the two patient groups most frequently treated. Participants were also asked to report the quantity of simple, prefabricated and customised FOs prescribed per week, and the extent of free choice they had when prescribing FOs.

The second section was designed to elicit the type of FO most frequently prescribed for patients with 26 pre-selected presentations and medical conditions affecting the back and lower limb, which included the following: back pain, hip pain, knee pain, patellofemoral pain, shin splints/poster-medial leg pain, ankle pain, Achilles tendonitis, rearfoot pain, plantar heel pain/plantar fasciitis, peroneal tendonitis, tibialis posterior tendon dysfunction, midfoot osteoarthritis, forefoot pain/metatarsalgia, Morton’s neuroma, 1st metatarsophalangeal joint osteoarthritis, diabetes with and without peripheral neuropathy, non-inflammatory musculoskeletal disease (e.g. osteoarthritis), seronegative arthritis, connective tissue disease, gout, neurological diseases, neuromuscular conditions, and falls prevention in older adults. The survey also captured data relating to early and established rheumatoid arthritis, the results of which will be included in a future publication. An electronic survey technique was used, utilising the Bristol Online Survey website (http://onlinesurveys.ac.uk) to enable international completion.

### Terminology

In the absence of universally agreed definitions of foot orthosis (FO) types, FOs were grouped into three categories. Simple FOs were considered as flat insoles with or without padding to accommodate painful areas or lesions. Prefabricated FOs were considered as devices made to a generic foot shape, contoured for the arch, and included modular prefabricated FOs that can be altered by clinicians (e.g. by the addition of heel posting, wedges, pads or top covers). Customised FOs were considered as devices manufactured for a specific person based on a 3D impression or computerised image of that person’s foot, and produced using computer aided device/manufacturing (CAD/CAM) or more traditional manufacturing techniques (e.g. foam impression box or plaster of Paris cast).

### Participants

Participants were invited to complete the anonymous online survey via professional bodies, national professional e-newsletters, special interest groups, discussion forums, and professional publications, across the UK, Australia and New Zealand. These countries were selected due to similar education and broadly comparable scopes of clinical practice in place. Data from podiatrists practising in any other countries who participated in the survey were excluded from the main analysis due to potential differences in education and scope of practice, but were presented as supplementary data. The survey was also promoted at local and regional meetings during the study period. To be eligible to complete the survey, participants had to be registered podiatrists, able to access the survey online, and understand written English. As the open invitation survey distribution method was designed to maximise participation, there was no denominator available to estimate the response rate.

### Analysis

Survey data was analysed with IBM SPSS v 21 (Armonk, NY: IBM Corp) using descriptive statistics. All data were described as mean (SD) for continuous data and n (%) for categorical data.

## Results

### Demographics

Two hundred and sixty-four podiatrists practising in 19 countries completed the survey. Of these, 147 (56%) were female. Respondents qualified between 1968 and 2016 with a mean (SD) of 16.9 (11.8) years since qualifying. Respondents practising in England, Scotland, Wales and Northern Ireland were combined as the UK. The majority of respondents practised in the UK (47%, *n* = 124), Australia (30%, *n* = 79) and New Zealand (12%, *n* = 32). Results from respondents practising outside these three countries (11%, *n* = 29) are not detailed but an overview is presented as supplementary data (see Additional file 2).

An overview of the type of health sector in which respondents in each country worked is shown in Table 1. Comparisons between the public sector and private practice were not made among respondents in Australia or New Zealand due to the limited number of respondents working solely in the public sector.

**Table 1:** Type of sector in which clinical time is spent

Country of practice	Solely public sector	Solely private practice	Combination
UK	42 (34%)	44 (35%)	38 (31%)
Australia	3 (4%)	64 (81%)	12 (15%)
New Zealand	2 (6%)	14 (44%)	16 (50%)

**Fig. 1:**
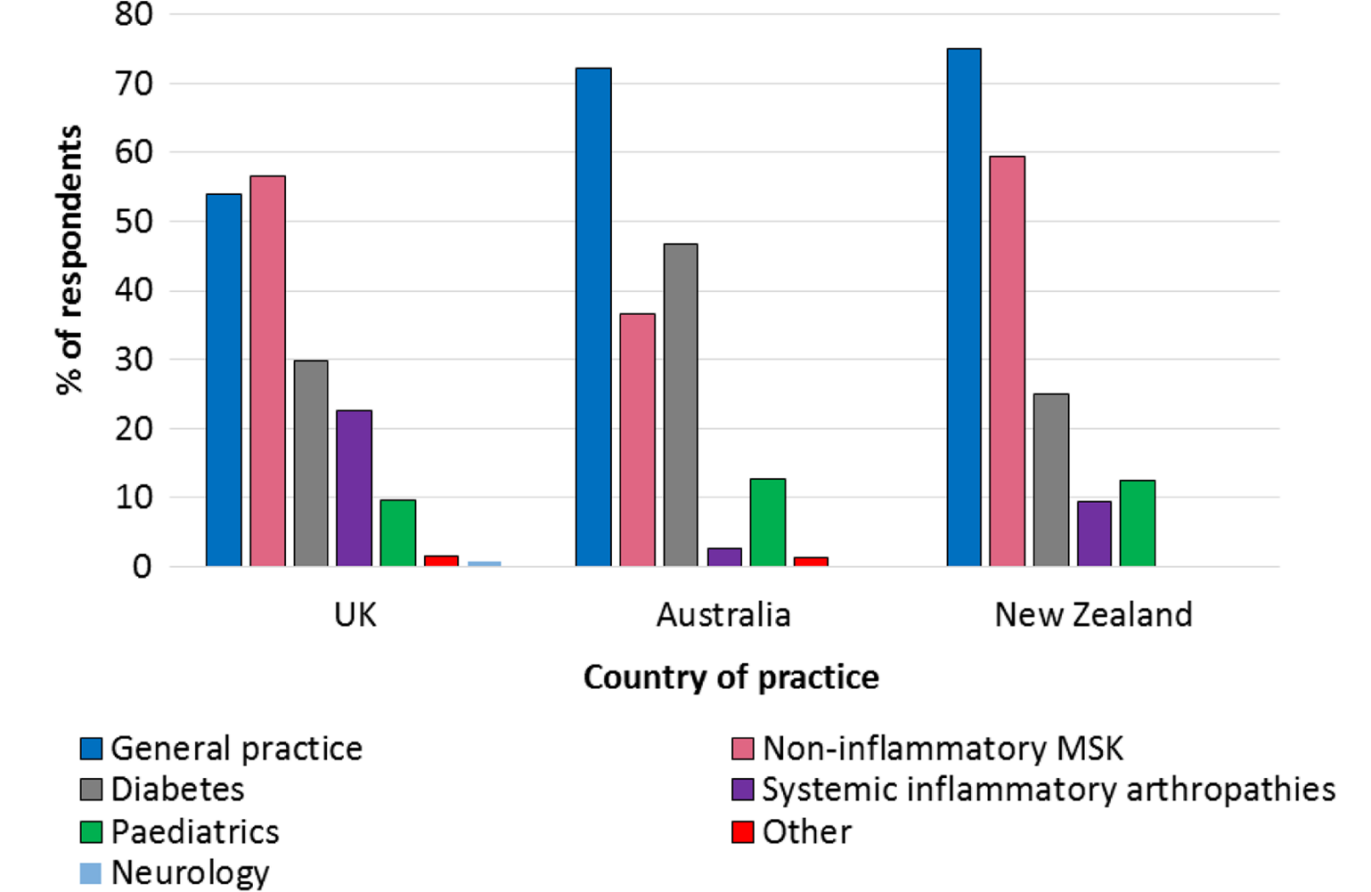
Most frequently treated patient groups

**Fig. 2:**
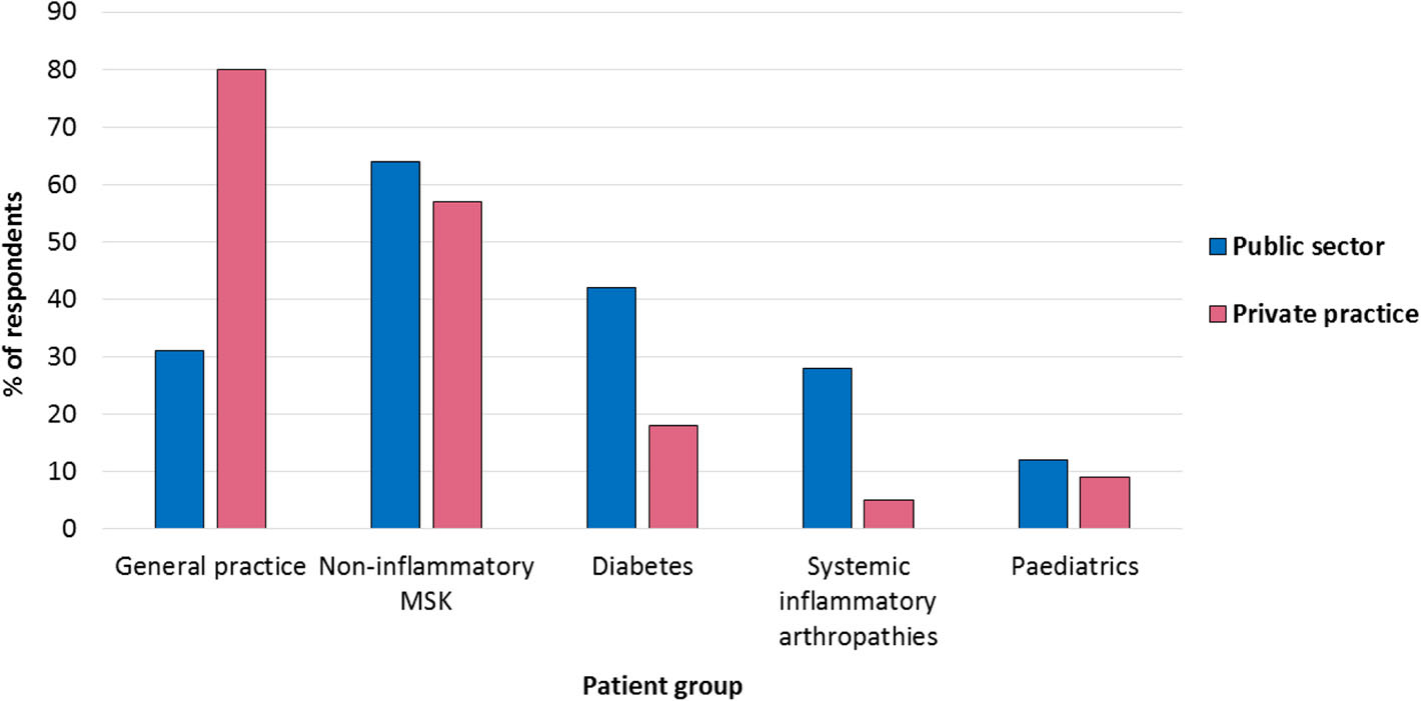
Most frequently treated patient groups in the UK by working sector

UK respondents most frequently treated people with non-inflammatory musculoskeletal conditions (Fig. 1). However, UK respondents working in private practice most frequently treated general practice/core podiatry patients (providing routine foot care, such as nail care and callus debridement) (Fig. 2). Respondents in Australia and New Zealand also most frequently treated general practice/core podiatry patients (Fig. 1).

### Quantity and type of FOs prescribed

**Fig. 3:**
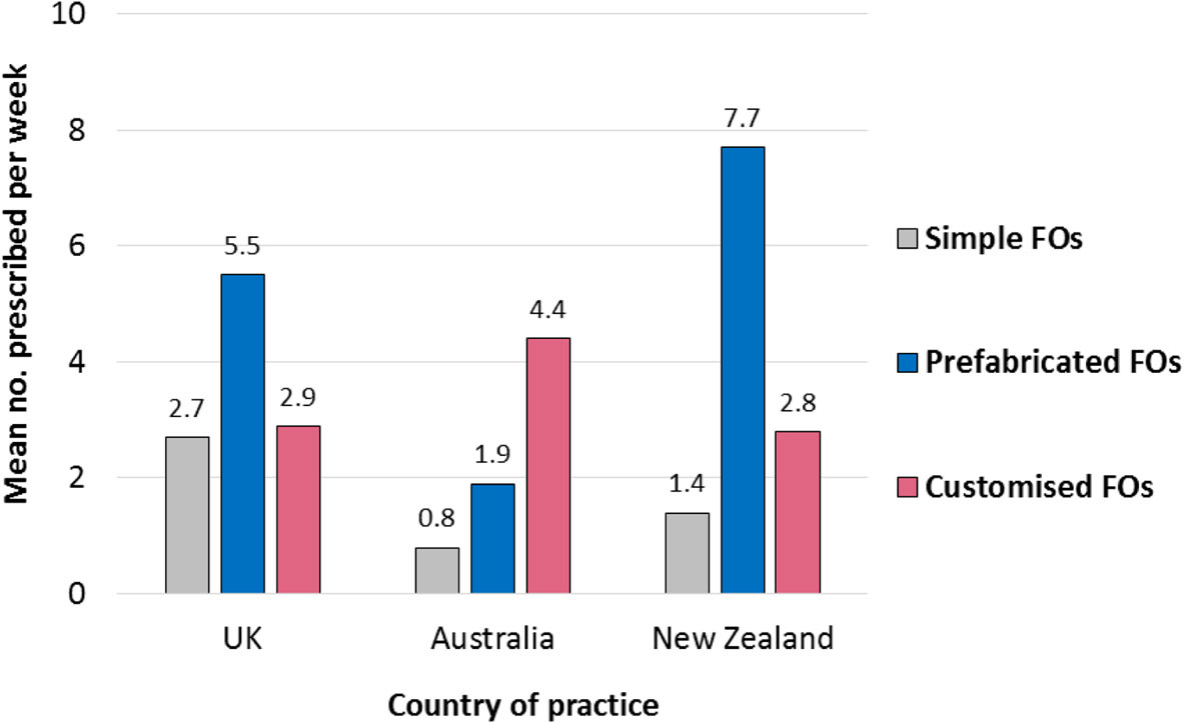
Most frequently prescribed FO types

Overall, prescription rates were higher amongst respondents in New Zealand and the UK, compared to Australian respondents (Fig. 3). Respondents practising in the UK prescribed more prefabricated FOs per week than customised or simple FOs. UK respondents prescribed the highest mean number of simple FOs per week compared to any other country (Fig. 3). UK respondents working in the public sector prescribed more prefabricated FOs per week than other FO types and over three times as many prefabricated FOs per week as those working in private practice (Table 2), whilst the provision of customised FOs was similar in both sectors. Among UK respondents working in private practice, more customised FOs were prescribed than prefabricated FOs and simple FOs, although differences were small (Table 2).

**Table 2:** Prescription habits among UK podiatrists by working sector

	Public sector (*n* = 42)			Private sector (*n* = 44)		
	Simple FOs	PFFOs	CFFOs	Simple FOs	PFFOs	CFFOs
Mean (SD) Range (per week)	2.8 (2.9) 0–10	8.2 (7.8) 0–30	2.2 (6.3) 0–40	1.5 (2.0) 0–10	2.4 (3.2) 0–15	2.6 (4.0) 0–15
No. (%) of respondents not prescribing FO type	7 (16.7%)	2 (4.8%)	14 (33.3%)	4 (9.1%)	6 (13.6%)	5 (11.4%)
No. (%) of respondents who had free choice when prescribing	12 (34%)	9 (25%)	11 (28%)	37 (93%)	34 (89%)	33 (85%)

*PFFOs prefabricated functional foot orthoses, CFFOs customised functional foot orthoses*

Although respondents in Australia prescribed fewer FOs per week than those in the UK and New Zealand, they had a higher mean number of customised FO prescriptions. They also prescribed more customised FOs per week than prefabricated FOs or simple FOs (Fig. 3). Respondents in New Zealand prescribed more prefabricated FOs per week than customised FOs or simple FOs, and more prefabricated FOs than those in Australia and the UK.

When asked about simple FOs, 16 (13%) respondents in the UK stated that they did not prescribe these devices. Similarly, 13 (17%) Australian respondents, and seven (22%) New Zealand respondents, did not prescribe simple FOs.

When asked about prefabricated FOs, 13 (11%) UK respondents indicated that they did not prescribe these devices. Similarly, four (5%) Australian respondents, and one (3%) New Zealand respondent indicated they did not prescribe prefabricated FOs.

When asked about customised FOs, 25 (20%) UK respondents stated that they did not prescribe these devices. In contrast, two (3%) respondents practising in Australia, and none (0%) of the respondents practising in New Zealand, stated they did not prescribe customised FOs.

### Extent of choice when prescribing

**Fig. 4:**
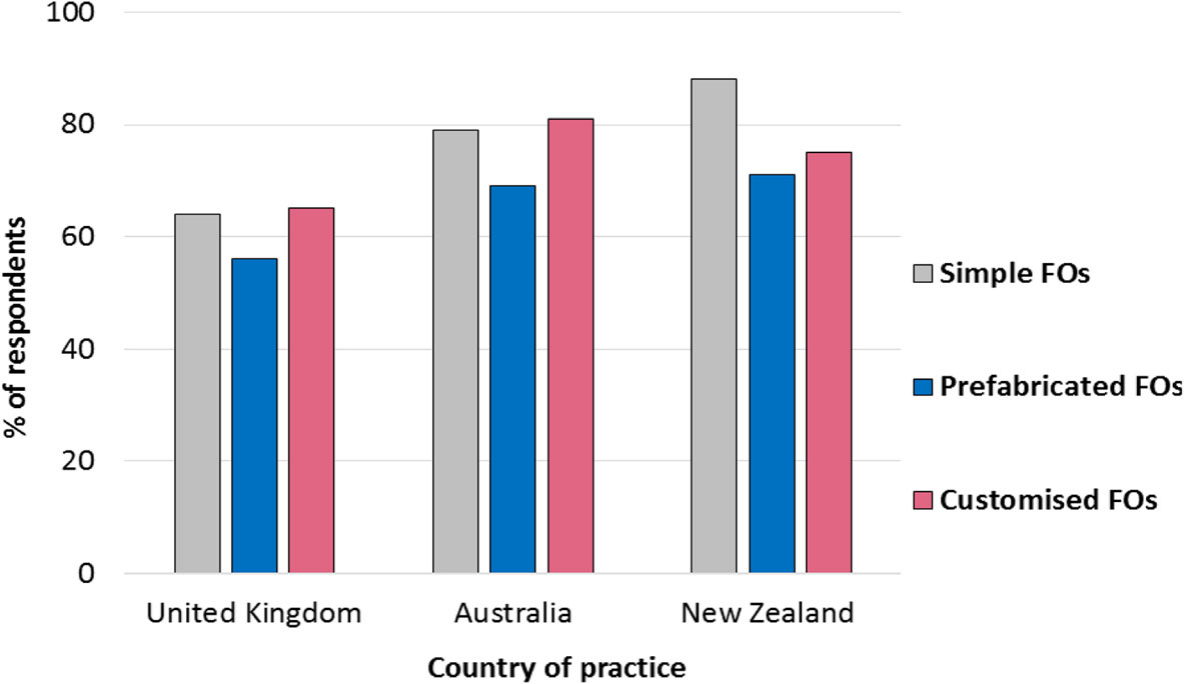
Percentage of respondents with free choice when prescribing FOs

Respondents in the UK had the least choice of which FO type (simple, prefabricated or customised) to prescribe, compared to respondents in Australia and New Zealand, and were more likely to have to select from a pre-determined list or stock (Fig. 4). Respondents in New Zealand had more choice than those in Australia when prescribing prefabricated FOs, while Australian respondents had more choice than those in New Zealand when prescribing customised FOs (Fig. 4). UK respondents working in the public sector had less free choice when prescribing all FO types than those working in private practice (Table 2).

### FO prescription habits for specific presentations and conditions

**Fig. 5:**
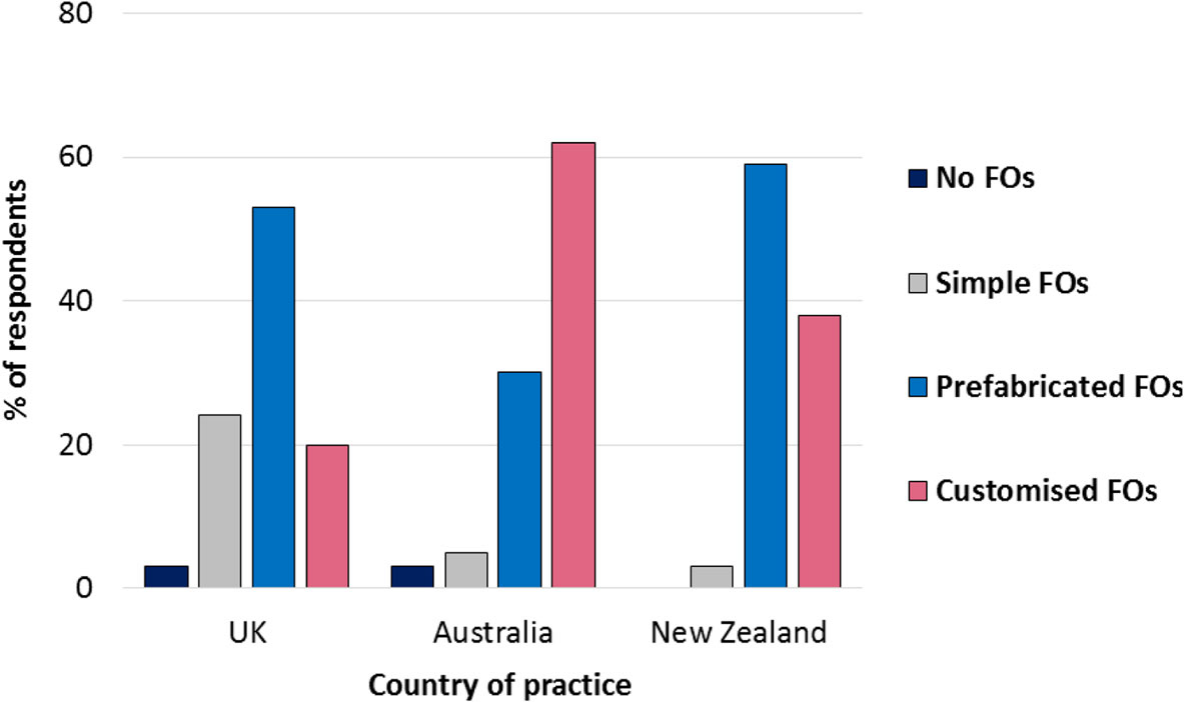
Types of FOs prescribed for 1st metatarsophalangeal joint osteoarthritis

Table 3 illustrates the range of FO prescription choices made among respondents from the UK, Australia and New Zealand for specific presentations and conditions. Overall, respondents practising in the UK and New Zealand indicated they were most likely to prescribe prefabricated FOs for the majority of presentations and conditions, whilst respondents in Australia indicated they were most likely to prescribe customised FOs. Fig. 5 illustrates the prescription pattern for 1st metatarsophalangeal joint osteoarthritis among respondents who treat the condition in practice. This pattern differed for seronegative inflammatory arthritis, connective tissue disease, neurological disorders and neuromuscular conditions among respondents in the UK; forefoot pain, Morton’s neuroma, gout, connective tissue disease and falls prevention among those in Australia; and tibialis posterior tendon dysfunction among those in New Zealand (Table 3).

**Table 3:** International prescription habits for specific presentations/conditions

	UK (*n* = 124)					Australia (*n* = 79)					New Zealand (*n* = 32)				
	Not treated	No FOs	Simple FOs	PFFOs	CFFOs	Not treated	No FOs	Simple FOs	PFFOs	CFFOs	Not treated	No FOs	Simple FOs	PFFOs	CFFOs
1. Back pain	40 (32.3%)	12 (9.7%)	8 (6.5%)	45 (36.3%)	19 (15.3%)	15 (19%)	3 (3.8%)	3 (3.8%)	19 (24.1%)	39 (49.4%)	12 (37.5%)	2 (6.3%)	0 (0%)	14 (43.8%)	4 (12.5%)
2. Hip pain	34 (27.4%)	11 (8.9%)	7 (5.6%)	51 (41.1%)	21 (16.9%)	11 (13.9%)	9 (11.4%)	1 (1.3%)	18 (22.8%)	40 (50.6%)	7 (21.9%)	0 (0%)	0 (0%)	21 (65.6%)	4 (12.5%)
3. Knee pain	19 (15.3%)	4 (3.2%)	9 (7.3%)	75 (60.5%)	17 (13.7%)	3 (3.8%)	1 (1.3%)	1 (1.3%)	25 (31.6%)	49 (62%)	3 (9.4%)	0 (0%)	1 (3.1%)	24 (75%)	4 (12.5%)
4. Patello-femoral pain	26 (21%)	5 (4%)	6 (4.8%)	71 (57.3%)	16 (12.9%)	3 (3.8%)	3 (3.8%)	0 (0%)	25 (31.6%)	48 (60.8%)	2 (6.3%)	0 (0%)	1 (3.1%)	26 (81.3%)	3 (9.4%)
5. Shin splints	17 (13.7%)	6 (4.8%)	8 (6.5%)	74 (59.7%)	19 (15.3%)	2 (2.5%)	1 (1.3%)	0 (0%)	28 (35.4%)	48 (60.8%)	1 (3.1%)	1 (3.1%)	0 (0%)	23 (71.9%)	7 (21.9%)
6. Ankle pain	9 (7.3%)	6 (4.8%)	11 (8.9%)	70 (56.5%)	28 (22.6%)	1 (1.3%)	4 (5.1%)	1 (1.3%)	22 (27.8%)	51 (64.6%)	0 (0%)	0 (0%)	0 (0%)	24 (75%)	8 (25%)
7. Achilles tendonitis	10 (8.1%)	7 (5.6%)	17 (13.7%)	76 (61.3%)	14 (11.3%)	1 (1.3%)	5 (6.3%)	8 (10.1%)	28 (35.4%)	37 (46.8%)	0 (0%)	4 (12.5%)	0 (0%)	24 (75%)	4 (12.5%)
8. Rearfoot pain	8 (6.5%)	4 (3.2%)	17 (13.7%)	74 (59.7%)	21 (16.9%)	2 (2.5%)	1 (1.3%)	0 (0%)	32 (40.5%)	44 (55.7%)	0 (0%)	1 (3.1%)	0 (0%)	23 (71.9%)	8 (25%)
9. Plantar heel pain / plantar fasciitis	5 (4%)	5 (4%)	23 (18.5%)	76 (61.3%)	15 (12.1%)	2 (2.5%)	1 (1.3%)	0 (0%)	35 (44.3%)	41 (51.9%)	0 (0%)	0 (0%)	0 (0%)	25 (78.1%)	7 (21.9%)
10. Peroneal tendonitis	13 (10.5%)	5 (4%)	13 (10.5%)	64 (51.6%)	29 (23.4%)	3 (3.8%)	5 (6.3%)	5 (6.3%)	22 (27.8%)	44 (55.7%)	0 (0%)	0 (0%)	0 (0%)	25 (78.1%)	7 (21.9%)
11. Tibialis posterior tendon dysfunction	10 (8.1%)	1 (0.8%)	11 (8.9%)	58 (46.8%)	44 (35.5%)	2 (2.5%)	1 (1.3%)	0 (0%)	16 (20.3%)	60 (75.9%)	0 (0%)	0 (0%)	0 (0%)	13 (40.6%)	19 (59.4%)
12. Midfoot pain/OA	9 (7.3%)	1 (0.8%)	19 (15.3%)	54 (43.5%)	41 (33.1%)	2 (2.5%)	1 (1.3%)	2 (2.5%)	33 (41.8%)	41 (51.9%)	0 (0%)	0 (0%)	1 (3.1%)	18 (56.3%)	13 (40.6%)
13. Forefoot pain/meta-tarsalgia	7 (5.6%)	4 (3.2%)	44 (35.5%)	50 (40.3%)	19 (15.3%)	3 (3.8%)	1 (1.3%)	11 (13.9%)	36 (45.6%)	28 (35.4%)	0 (0%)	0 (0%)	4 (12.5%)	22 (68.8%)	6 (18.8%)
14. Morton’s neuroma	8 (6.5%)	5 (4%)	45 (36.3%)	47 (37.9%)	19 (15.3%)	3 (3.8%)	5 (6.3%)	14 (17.7%)	30 (38%)	27 (34.2%)	0 (0%)	1 (3.1%)	8 (25%)	19 (59.4%)	4 (12.5%)
15. 1st MTPJ OA	5 (4%)	4 (3.2%)	28 (22.6%)	63 (50.8%)	24 (19.4%)	2 (2.5%)	2 (2.5%)	4 (5.1%)	23 (29.1%)	48 (60.8%)	0 (0%)	0 (0%)	1 (3.1%)	19 (59.4%)	12 (37.5%)
16. Diabetes without peripheral neuropathy	9 (7.3%)	19 (15.3%)	29 (23.4%)	48 (38.7%)	19 (15.3%)	3 (3.8%)	25 (31.6%)	9 (11.4%)	23 (29.1%)	19 (24.1%)	4 (12.5%)	2 (6.3%)	2 (6.3%)	22 (68.8%)	2 (6.3%)
17. Diabetes with peripheral neuropathy	15 (12.1%)	11 (8.9%)	29 (23.4%)	28 (22.6%)	41 (33.1%)	5 (6.3%)	14 (17.7%)	13 (16.5%)	17 (21.5%)	30 (38%)	5 (15.6%)	1 (3.1%)	5 (15.6%)	15 (46.9%)	6 (18.8%)
18. Non-inflammatory musculoskeletal disease	5 (4%)	4 (3.2%)	25 (20.2%)	72 (58.1%)	18 (14.5%)	2 (2.5%)	3 (3.8%)	5 (6.3%)	32 (40.5%)	37 (46.8%)	0 (0%)	1 (3.1%)	2 (6.3%)	23 (71.9%)	6 (18.8%)
19. Sero-negative inflammatory arthritis	22 (17.7%)	10 (8.1%)	11 (8.9%)	42 (33.9%)	39 (31.5%)	11 (13.9%)	7 (8.9%)	4 (5.1%)	20 (25.3%)	37 (46.8%)	8 (25%)	0 (0%)	5 (15.6%)	12 (37.5%)	7 (21.9%)
20. Gout	20 (16.1%)	33 (26.6%)	23 (18.5%)	37 (29.8%)	11 (8.9%)	10 (12.7%)	28 (35.4%)	7 (8.9%)	18 (22.8%)	16 (20.3%)	8 (25%)	4 (12.5%)	0 (0%)	17 (53.1%)	3 (9.4%)
21. Connective tissue disease	28 (22.6%)	24 (19.4%)	20 (16.1%)	28 (22.6%)	24 (19.4%)	16 (20.3%)	19 (24.1%)	3 (3.8%)	22 (27.8%)	19 (24.1%)	6 (18.8%)	2 (6.3%)	4 (12.5%)	16 (50%)	4 (12.5%)
22. Neurological diseases	27 (21.8%)	21 (16.9%)	18 (14.5%)	27 (21.8%)	31 (25%)	12 (15.2%)	15 (19%)	2 (2.5%)	15 (19%)	35 (44.3%)	7 (21.9%)	2 (6.3%)	2 (6.3%)	11 (34.4%)	10 (31.3%)
23. Neuro-muscular conditions	27 (21.8%)	22 (17.7%)	19 (15.3%)	25 (20.2%)	31 (25%)	10 (12.7%)	20 (25.3%)	4 (5.1%)	17 (21.5%)	28 (35.4%)	8 (25%)	3 (9.4%)	3 (9.4%)	10 (31.3%)	8 (25%)
24. Falls prevention in older adults	26 (21%)	22 (17.7%)	19 (15.3%)	46 (37.1%)	11 (8.9%)	7 (8.9%)	21 (26.6%)	4 (5.1%)	28 (35.4%)	19 (24.1%)	8 (25%)	5 (15.6%)	0 (0%)	15 (46.9%)	4 (12.5%)

*PFFOs prefabricated functional foot orthoses, CFFOs customised functional foot orthoses, MTPJ metatarsophalangeal joint, OA osteoarthritis*

**Fig. 6:**
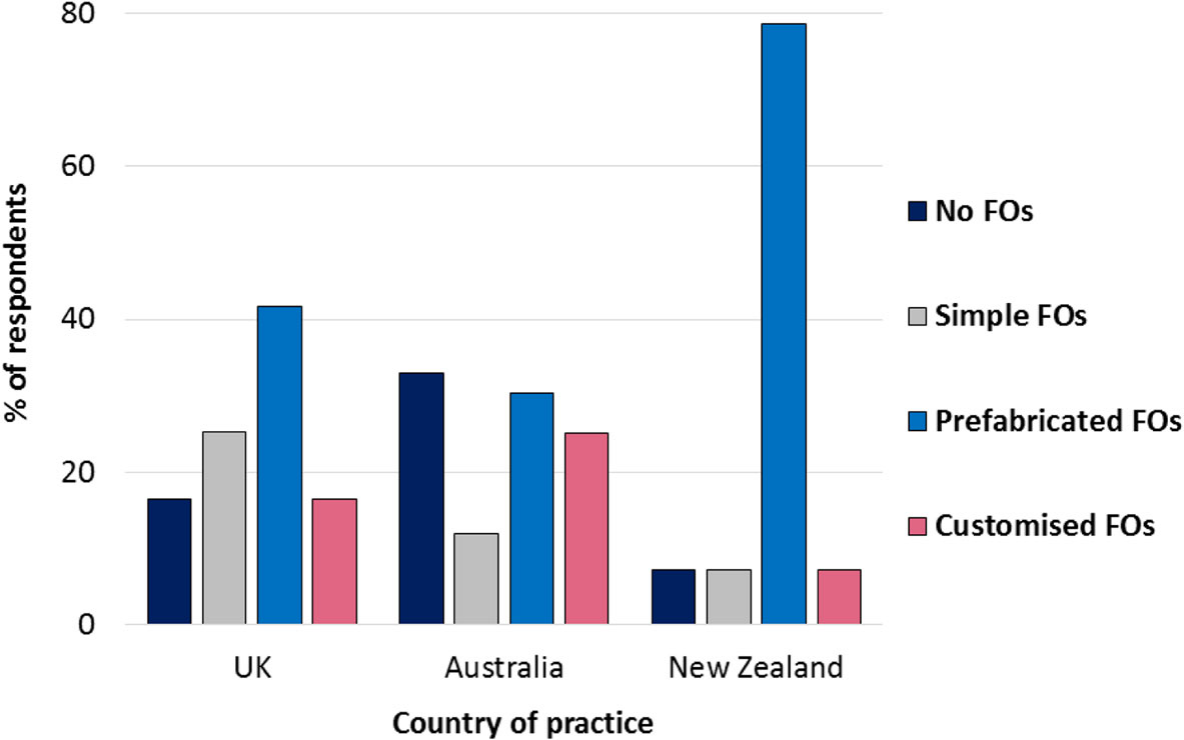
Types of FOs prescribed for diabetes without peripheral neuropathy

**Fig. 7:**
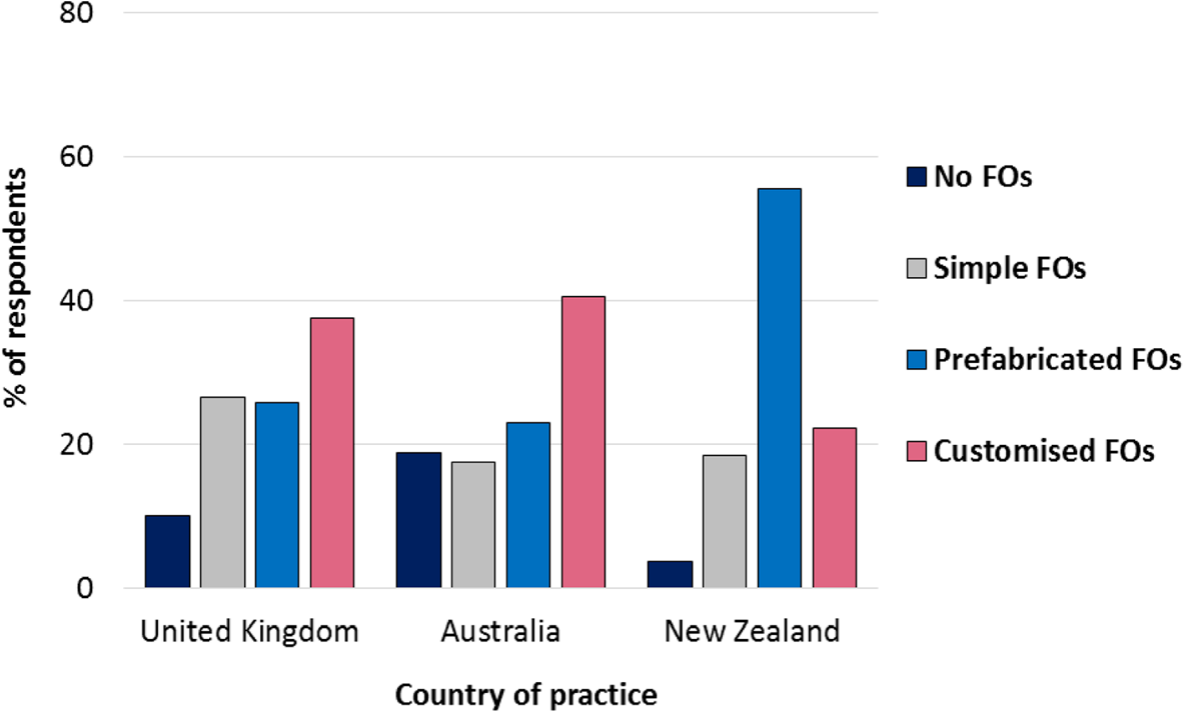
Types of FOs prescribed for diabetes with peripheral neuropathy

Figures 6 and 7 illustrate FO prescription choices for diabetes without and with peripheral neuropathy, respectively, among respondents who confirmed that they treat the condition in practice. When treating diabetes without peripheral neuropathy, respondents in the UK were most likely to prescribe prefabricated FOs, whereas customised FOs were the most frequent prescription choice for diabetes with peripheral neuropathy. Respondents in Australia reported a range of FO prescription choices for diabetes without peripheral neuropathy, and were less likely to prescribe any FO for this condition compared to prescribing simple, prefabricated or customised FOs. In contrast, Australian respondents were most likely to prescribe customised FOs for diabetes with peripheral neuropathy. Respondents in New Zealand were most likely to prescribe prefabricated FOs for people with diabetes regardless of the presence of peripheral neuropathy, although they were three times more likely to prescribe customised FOs for diabetes with peripheral neuropathy than without.

## Discussion

This study explored and compared the types of FOs currently prescribed by podiatrists in the UK, Australia and New Zealand, and reports whether the type of FO prescribed varies between a range of presentations and conditions affecting the back and lower limb. Differences in the frequency of FO types prescribed per week were identified between countries. We found that a higher mean quantity of customised FOs compared to other FO types were prescribed by Australian respondents, and a higher mean quantity of prefabricated FOs were prescribed by New Zealand respondents compared to Australian respondents. This finding is similar to a previous study by Landorf et al. [7], in which Australian podiatrists were found to more frequently prescribe customised FOs, but podiatrists in New Zealand were three times more likely than those in Australia to prescribe prefabricated FOs. In our study, respondents in the UK demonstrated similar weekly FO prescription habits to those in New Zealand, as more prefabricated FOs were prescribed than other FO types, and the mean quantity of customised FOs prescribed per week was almost identical. We found that 11% of UK respondents did not prescribe prefabricated FOs and 20% did not prescribe customised FOs. These UK findings are similar to work conducted by Nester et al. [22], where 7% of the UK clinicians (podiatrists, physiotherapists and orthotists) surveyed did not use prefabricated FOs and 25% did not use customised FOs in practice. Despite these similarities in our UK data, our sample consisted entirely of podiatrists, therefore results should not be extrapolated to other professions.

Differences in FO prescription habits found between countries may be linked to the sector in which respondents work. The majority (81%) of Australian respondents worked solely in private practice, compared to 35% in the UK and 44% in New Zealand. Findings indicated that although respondents in Australia prescribed far fewer FOs overall, they prescribed more customised FOs, which previous studies have shown to be more expensive to produce than prefabricated and simple FOs [3, 16]. Differences in the types of FOs prescribed between UK respondents working in the public sector and those working in private practice were also identified. Respondents in the UK public sector prescribed more simple and prefabricated FOs compared to those working in private practice, whilst respondents in UK private practice prescribed more customised FOs. These differences may reflect NHS cost savings, which present a fundamental issue within UK public sector healthcare services [23]. The increased number of customised FOs prescribed in Australia, compared to the UK and New Zealand, may also be a result of differences in healthcare systems and subsequent health insurance schemes that are in place.

Our study reveals new insights into which FOs podiatrists prescribe according to the presentation or condition affecting the lower limb. Differences in FO prescription habits between countries were evident for specific presentations and conditions. Respondents in the UK and Australia indicated a preference for customised FOs when treating diabetes with peripheral neuropathy, despite a lack of robust evidence supporting these clinical decisions. For example, in one randomised trial customised FOs were found to be no better than prefabricated insoles in reducing the risk of diabetic neuropathic ulceration and the authors suggested that prefabricated insoles should be prescribed instead of customised devices where appropriate [9]. However, some international guidelines currently recommend considering custom-made insoles when a patient with diabetes has a foot deformity or the skin shows pre-ulcerative signs [24], which may influence prescription habits. Respondents in New Zealand were most likely to prescribe prefabricated FOs for diabetes with and without peripheral neuropathy compared to other FO types. Although, the higher rate of provision of customised FO for people with diabetes with peripheral neuropathy, compared to without neuropathy, suggests that the presence of neuropathy also influenced prescription habits among New Zealand respondents.

Additionally, the majority of respondents who treated back pain and hip pain in practice prescribed FOs. Respondents in the UK and New Zealand prescribed more prefabricated FOs for back pain and hip pain, whilst those in Australia prescribed more customised FOs. This is despite an absence of evidence for the effectiveness of any FOs for these presentations [25], and indeed the NICE guidelines explicitly state that FOs should not be used in the treatment of lower back pain [26]. However, as the survey was designed to recruit podiatrists who prescribed FOs, rather than who treated specific conditions, results may not be representative of the wider population of podiatrists.

Findings from this study must be considered in the context of its limitations. Firstly, the open invitation method of survey distribution did not allow a denominator population of podiatrists to be determined accurately. Although accurate figures are available for the number of registered podiatrists in each country, not all of these are members of their professional body, and professional bodies will not have up to date email addresses for all members. Therefore formal calculation of a response rate is not possible. Secondly, despite efforts to maximise recruitment with this method, the sample size was lower than hoped for, which may limit the generalisability of our findings and we note that there are 4800 registered podiatrists in Australia, 12,700 in the UK and 450 in New Zealand. However, the survey did elicit detailed information about FO prescription habits for multiple conditions, leading to a compromise between the depth of information and breadth of population covered. The survey required participants to select the type of FO that they would prescribe most frequently for a range of common presentations and conditions affecting the back and lower limb, and as such, provides a benchmark of contemporary practice, allowing clinicians to reflect on their current FO prescription habits against national and international patterns.

Some respondents indicated in free text within the comments section at the end of the survey that they would not necessarily prescribe FOs based on the presence of a certain presentation or condition. It was stated that the decision to prescribe a FO, and the type of FO prescribed, would depend on the individual and whether they were symptomatic or not, factoring in pain levels, foot shape, deformity, and joint range of motion. Several participants also identified that the type of FO prescribed for a certain condition would depend on the patient’s financial situation or their footwear. Gaining deeper insight into the clinical decision-making processes that underpin FO prescription was beyond the scope of this survey, but clinicians’ choices in relation to prescribing FOs for specific lower limb pathologies across different countries and healthcare sectors is certainly an area for future research.

## Conclusions

This study has identified that FO prescription habits vary depending on the country of practice, the healthcare sector in which practitioners work, and the condition targeted. Australian respondents, the majority of whom worked in private practice, prescribed far fewer devices overall, but when they did prescribe, they were more likely to prescribe customised FOs. In contrast, prefabricated FOs were more frequently prescribed in the UK and New Zealand, where there was more variation in working sector. FO prescription habits for diabetes differed according to whether or not peripheral neuropathy was present. An increased percentage of respondents prescribed customised FOs for diabetes with peripheral neuropathy compared to diabetes without peripheral neuropathy, independent of country of practice. These findings provide insights into contemporary clinical practice.

## Additional files

Additional file 1: Copy of FO survey. (PDF 120 kb)

Additional file 2: Data from countries outside of the UK, Australia and New Zealand. (DOCX 16 kb)

## Abbreviations

3D: 3-dimensional
CAD/CAM: Computer aided device/computer aided manufacture
FOs: Foot orthoses
NHS: National Health Service
SD: Standard deviation
UK: United Kingdom

## References

[1] Menz HB (2009). Foot orthoses: how much customisation is necessary?. J Foot Ankle Res.

[2] Banwell HA, Mackintosh S, Thewlis D, Landorf KB (2014). Consensus-based recommendations of Australian podiatrists for the prescription of foot orthoses for symptomatic flexible pes planus in adults. J Foot Ankle Res.

[3] Redmond AC, Landorf KB, Keenan AM (2009). Contoured, prefabricated foot orthoses demonstrate comparable mechanical properties to contoured, customised foot orthoses: a plantar pressure study. J Foot Ankle Res..

[4] Landorf KB, Keenan AM (2000). Efficacy of foot orthoses. What does the literature tell us?. J Am Podiatr Med Assoc.

[5] Landorf KB, Keenan AM, Herbert RD (2006). Effectiveness of foot orthoses to treat plantar fasciitis: a randomized trial. Arch Intern Med.

[6] Hennessy K, Woodburn J, Steultjens MP (2012). Custom foot orthoses for rheumatoid arthritis: a systematic review. Arthritis Care Res (Hoboken).

[7] Landorf K, Keenan AM, Rushworth RL (2001). Foot orthosis prescription habits of Australian and New Zealand podiatric physicians. J Am Podiatr Med Assoc.

[8] Redmond AC, Alcacer-Pitarch B, Gray J, Denton CP, Herrick A, Navarro-Coy N (2013). Simple insoles for managing foot problems in people with ssc: the PISCES randomised controlled trial. Rheumatology.

[9] Paton JS, Stenhouse EA, Bruce G, Zahra D, Jones RB (2012). A comparison of customised and prefabricated insoles to reduce risk factors for neuropathic diabetic foot ulceration: a participant-blinded randomised controlled trial. J Foot Ankle Res.

[10] Woodburn J, Barker S, Helliwell PS (2002). A randomized controlled trial of foot orthoses in rheumatoid arthritis. J Rheumatol.

[11] Woodburn J, Helliwell PS, Barker S (2003). Changes in 3D joint kinematics support the continuous use of orthoses in the management of painful rearfoot deformity in rheumatoid arthritis. J Rheumatol.

[12] Bonanno DR, Murley GS, Munteanu SE, Landorf KB, Menz HB (2017). Effectiveness of foot orthoses for the prevention of lower limb overuse injuries in naval recruits: a randomised controlled trial. Br J Sports Med.

[13] NICE. National Institute for Health and Care Excellence. Rheumatoid arthritis in adults: management. Clinical guideline [CG79] 2009. Available from: https://www.nice.org.uk/guidance/cg79/resources/rheumatoid-arthritis-in-adults-management-pdf-975636823525. Accessed 21 October 2017.

[14] NICE. National Institute for Health and Care Excellence. Osteoarthritis: care and management 2014. Available from: https://www.nice.org.uk/guidance/cg177/resources/osteoarthritis-care-and-management-pdf-35109757272517 Accessed 20 July 2017.

[15] NICE. National Institute for Health and Care Excellence. Diabetic foot problems: prevention and management. Clinical guideline [NG19] 2015. Available from: https://www.nice.org.uk/guidance/ng19/resources/diabetic-foot-problems-prevention-and-management-pdf-1837279828933 Accessed 2 August 2017

[16] Rome K, Clark H, Gray J, McMeekin P, Plant M, Dixon J (2017). Clinical effectiveness and cost-effectiveness of foot orthoses for people with established rheumatoid arthritis: an exploratory clinical trial. Scand J Rheumatol.

[17] Backhouse MR, Bowen CJ (2007). Effect of prefabricated and custom orthoses on plantar loading of the first metatarsophalangeal joint during gait. Br J Pod.

[18] Hutton J, Hurry M (2009). Orthotic Service in the NHS: Improving Service Provision.

[19] Guldemond NA, Leffers P, Schaper NC, Sanders AP, Nieman FH, Walenkamp GH (2005). Comparison of foot orthoses made by podiatrists, pedorthists and orthotists regarding plantar pressure reduction in the Netherlands. BMC Musculoskelet Disord.

[20] Chevalier T, Chockalingam N (2012). Effects of foot orthoses: how important is the practitioner?. Gait Posture.

[21] Menz HB, Allan JJ, Bonanno DR, Landorf KB, Murley GS (2017). Custom-made foot orthoses: an analysis of prescription characteristics from an Australian commercial orthotic laboratory. J Foot Ankle Res.

[22] Nester CJ, Graham A, Martinez-Santos A, Williams AE, McAdam J, Newton V (2017). National profile of foot orthotic provision in the United Kingdom, part 1: practitioners and scope of practice. J Foot Ankle Res.

[23] The King's Fund. NHS Five Year Forward View. 2014. Available from: https://www.england.nhs.uk/wp-content/uploads/2014/10/5yfv-web.pdf. Accessed 14 December 2012.

[24] IWGDF. The International Working Group on the Diabetic Foot. Guidance on footwear and offloading 2015. Available from: http://iwgdf.org/guidelines/guidance-on-footwear-and-offloading-2015/. Accessed 4 May 2018.

[25] Chuter V, Spink M, Searle A, Ho A (2014). The effectiveness of shoe insoles for the prevention and treatment of low back pain: a systematic review and meta-analysis of randomised controlled trials. BMC Musculoskelet Disord.

[26] NICE. National Institute for Health and Care Excellence. Low back pain and sciatica in over 16s: assessment and management. Clinical guideline [NG59] 2016. Available from: https://www.nice.org.uk/guidance/ng59/resources/low-back-pain-and-sciatica-in-over-16s-assessment-and-management-pdf-1837521693637. Accessed 18 September 2018. 31841295

