# Supplementary material for: A survey of foot orthoses prescription habits amongst podiatrists in the UK, Australia and New Zealand
Source: J Foot Ankle Res. 2018 Nov 26;11:64. doi: 10.1186/s13047-018-0304-z (PMC6258496; doi:10.1186/s13047-018-0304-z)
Supplement: Supplementary file 1 — Copy of FO survey. (PDF 120 kb) [file 13047_2018_304_MOESM1_ESM.pdf]

# Foot Orthoses Survey

Dear Colleague,

Thank you for participating in this international survey that will help us to describe the types of foot orthoses (FOs) currently in use. This study is being led by three podiatrists: Dr Michael Backhouse from the University of Leeds, UK; A/Prof Karl Landorf from La Trobe University, Australia; and Prof Keith Rome from Auckland University of Technology, New Zealand. Your answers will help us to build a picture of the types of FOs prescribed for a variety of common conditions affecting the foot and ankle, and will inform future research relating to the prescription of FOs.

As well as contributing to this survey you also have the chance to **win an iPad mini.** The survey will take approximately 10 minutes to complete. We are interested in the views of **registered podiatrists** who prescribe FOs as part of their routine practice.

In this survey, the following definitions will be used:

1. **Simple FOs** refer to flat insoles with or without padding to accommodate painful areas or lesions.
2. **Prefabricated FOs** refer to orthoses that are made to a generic foot shape. They are contoured for the arch and include modular prefabricated orthoses that can be altered by clinicians (e.g. by the addition of posting, wedges, pads or top-covers).
3. **Customised orthoses** refer to orthoses that are manufactured for a specific person based on a 3D impression or computerised image of that person's foot. Custom FOs may be produced using CAD/CAM, or more traditional manufacturing techniques (e.g. foam impression box or plaster of paris cast).

Once you have completed the survey, you will not receive any follow-up communication from us and your participation will remain completely anonymous if you choose. However, if you leave your email address at the end of the survey, you will be entered into our competition to **win a free iPad mini.**

Australian Podiatrists can earn 1 hour CPD for completing the survey, although importantly you must retain a copy of your completed answers. Full instructions are at the end of the survey.

Click here to access the participant information page for this survey.

Thank you and best wishes,

Dr Michael Backhouse, University of Leeds, United Kingdom, A/Prof Karl Landorf, La Trobe University, Australia, Prof Keith Rome, Auckland University of Technology, New Zealand

This research is funded by the College of Podiatry, London, UK and the National Institute of Health Research, UK.

Ethical approval has been granted by the University of Leeds, UK.

# Section 1: Information about you and your practice

1. Are you

- ○ Male
- ○ Female
- ○ Other

2. In which year did you qualify?

[ ]

3. In which country do you practice? (please select all that apply)

- ☐ Australia
- ☐ Canada
- ☐ England
- ☐ New Zealand
- ☐ Northern Ireland
- ☐ Republic of Ireland
- ☐ Scotland
- ☐ Wales
- ☐ Other (please state)

3.a. If you selected Other, please specify:

[ ]

4. In which country did you undertake your primary podiatry qualification?

(please select one)

- ☐ Australia
- ☐ Canada
- ☐ England
- ☐ New Zealand
- ☐ Northern Ireland
- ☐ Republic of Ireland
- ☐ Scotland
- ☐ Wales
- ☐ Other

*4.a.* If you selected Other, please specify:

[ ]

*5.* Please state the percentage of your clinical time spent in the private/independent and public sectors. (Your response should total 100.)

| | percentage |
|---|---|
| public | |
| private/independent | |

*6.* Which groups of patients do you treat most frequently? (please select a maximum of two categories)

- ☐ Systemic inflammatory arthropathies (e.g. rheumatoid arthritis)
- ☐ Non-inflammatory musculoskeletal conditions / biomechanics
- ☐ Diabetes

- ☐ Neurology
- ☐ Paediatrics
- ☐ General practice / routine foot care / core podiatry
- ☐ Other (please state)

*6.a.* If you selected Other, please specify:

*7.* On average, how many pairs of FOs you prescribe each week are:

More info

| | number of pairs per week |
|---|---|
| Simple FO | |
| Prefabricated FO | |
| Customised FO | |

*8.* Do you have free choice of which **simple FOs** to prescribe in your practice?

More info

- ○ Yes, I have free choice
- ○ No, I must select from a pre-determined list or stock
- ○ I do not prescribe simple FOs

*9.* Do you have free choice of which **prefabricated FOs** to prescribe within your practice?

More info

- Yes, I have free choice
- No, I must select from a pre determined list or stock
- I do not prescribe prefabricated FOs

*10.* Do you have free choice of which **customised FOs** to prescribe within your practice?

More info

- Yes, I have free choice
- No, I must select from a pre determined list of prescription variables or materials
- I do not prescribe customised FOs

# Section 2: Information about the FOs you prescribe.

This section includes questions about the type of FOs that you prescribe for different conditions.

In this survey, the following definitions are used:

1. **Simple FOs** refer to flat insoles with or without padding to accommodate painful areas or lesions.
2. **Prefabricated FOs** refer to orthoses that are made to a generic foot shape. This includes modular prefabricated orthoses that can be altered by clinicians (e.g. by the addition of posting, wedges, pads or top-covers).
3. **Customised orthoses** refer to orthoses that are manufactured for a specific person based on a 3D impression or computerised image of that person's foot. Custom FOs may be produced using CAD/CAM, or more traditional manufacturing techniques (e.g. foam impression box or plaster of paris cast).

*11.* What type of FO are you most likely to prescribe for the following presentations?

More info

| | I do not treat patients with this condition | no FO's | simple FO's | prefabricated FO's | customised FO's |
|---|---|---|---|---|---|
| Back pain | ◯ | ◯ | ◯ | ◯ | ◯ |
| Hip pain | ◯ | ◯ | ◯ | ◯ | ◯ |
| Knee pain | ◯ | ◯ | ◯ | ◯ | ◯ |
| Patellofemoral pain | ◯ | ◯ | ◯ | ◯ | ◯ |
| Shin splints / poster-medial leg pain | ◯ | ◯ | ◯ | ◯ | ◯ |
| Ankle pain | ◯ | ◯ | ◯ | ◯ | ◯ |

| | | | | | |
|---|---|---|---|---|---|
| Achilles tendonitis / tendonosis | ○ | ○ | ○ | ○ | ○ |
| Rearfoot pain | ○ | ○ | ○ | ○ | ○ |
| Plantar heel pain / plantar fasciitis | ○ | ○ | ○ | ○ | ○ |
| Peroneal tendonitis | ○ | ○ | ○ | ○ | ○ |
| Tibialis posterior tendon dysfunction | ○ | ○ | ○ | ○ | ○ |
| Midfoot pain / midfoot osteoarthritis | ○ | ○ | ○ | ○ | ○ |
| Forefoot pain / metatarsalgia | ○ | ○ | ○ | ○ | ○ |
| Mortons neuroma | ○ | ○ | ○ | ○ | ○ |
| 1st MTPJ osteoarthritis | ○ | ○ | ○ | ○ | ○ |

*12.* What type of FO are you most likely to prescribe for the following specific conditions?

More info

| | I do not treat patients with this condition | no FO's | simple FO's | prefabricated FO's | customised FO's |
|---|---|---|---|---|---|
| Diabetes without peripheral neuropathy | ○ | ○ | ○ | ○ | ○ |

| | | | | | |
|---|---|---|---|---|---|
| Diabetes with peripheral neuropathy | ○ | ○ | ○ | ○ | ○ |
| Non-inflammatory musculoskeletal disease (e.g. osteoarthritis) | ○ | ○ | ○ | ○ | ○ |
| Early rheumatoid arthritis (i.e. less than 2 years) | ○ | ○ | ○ | ○ | ○ |
| Established rheumatoid arthritis | ○ | ○ | ○ | ○ | ○ |
| Seronegative inflammatory arthritis (e.g. psoriatic arthritis) | ○ | ○ | ○ | ○ | ○ |
| Gout | ○ | ○ | ○ | ○ | ○ |
| Connective tissue disease (e.g. lupus, scleroderma) | ○ | ○ | ○ | ○ | ○ |
| Neurological diseases (e.g. stroke) | ○ | ○ | ○ | ○ | ○ |
| Neuromuscular conditions (e.g. MS & Parkinson's) | ○ | ○ | ○ | ○ | ○ |
| Falls prevention in older adults | ○ | ○ | ○ | ○ | ○ |

*13.* The last section contains questions about prescribing FOs for people with rheumatoid arthritis.

○ I do not prescribe FOs for people with rheumatoid arthritis.

- I prescribe FOs for people with rheumatoid arthritis.

# Section 3. Rheumatoid Arthritis

This is the final section of the questionnaire and we would now like to ask you about the orthoses you use in people with **early and established rheumatoid arthritis**. Here we consider early rheumatoid arthritis to refer to those who have had RA for 2 years or less.

## Early Rheumatoid Arthritis

*14.* If you prescribe prefabricated FOs for people with early rheumatoid arthritis, please state the brand and model you use most frequently in the box below.

More info

*15.* Do you prescribe customised orthoses for patients with early rheumatoid arthritis?

More info

- no
- yes

# Early Rheumatoid Arthritis Customised Orthoses

*16.* When you prescribe customised FOs for patients with early rheumatoid arthritis, which method do you use most frequently to capture the 3D shape of the foot? (please choose one)

More info

- Plaster of paris
- Foam impression box
- Electronic scanning or imaging

*16.a.* Is this method . . .

- Weightbearing
- Non-weightbearing

*17.* When you prescribe customised FOs for people with early rheumatoid arthritis, what manufacturing techniques do you use most frequenty to manufacture the FOs? (please choose one)

More info

- Computer aided manufacture (e.g. additive manufacturing such as 3D printing, or direct milling)
- Traditional manufacturing techniques (e.g. vacuum forming)

*18.* When you prescribe customised insoles for people with early rheumatoid arthritis, what type of shell material do you specify most frequently? (please choose one)

More info

- Highly rigid (e.g. carbon fibre)
- Semi rigid (e.g. polypropylene)
- Semi flexible (e.g. high density EVA)
- Highly flexible / cushioning (e.g. medium or low density EVA)

*19.* When you prescribe customised insoles for people with early rheumatoid arthritis, what rearfoot posting do you specify most frequently? (please choose one)

More info

- None
- Intrinsic posting
- Highly rigid (e.g. acrylic / carbon fibre)
- Semi rigid (e.g. polypropylene)
- Semi flexible (e.g. high density EVA)
- Highly flexible/cushioning (e.g. medium or low density EVA)

*20.* When you prescribe customised insoles for early rheumatoid arthritis patients, what top cover materials do you specify? (please choose all that apply)

More info

- Minimal (e.g. leather / vinyl)
- Cushioning (e.g. Poron or similar polyurethane)
- Cushioning with specific modification to offload or cushion the forefoot
- Cushioning with specific modification to offload or cushion the midfoot
- Cushioning with specific modification to offload or cushion the rearfoot

# Established Rheumatoid Arthritis

*21.* If you prescribe prefabricated FOs for people with established rheumatoid arthritis, please state the brand and model you use most frequently in the box below. *Optional*

More info

*22.* Do you prescribe customised FOs for patients with established rheumaoid arthritis?

More info

- yes
- no

# Establish Rheumatoid Arthritis Customised FOs

23. When you prescribe customised FOs for patients with established rheumatoid arthritis, which method do you use most frequently to capture the 3D shape of the foot? (please choose one)

More info

- Plaster of paris
- Foam impression
- Electronic scanning or imaging

23.a. Is this method . . .

- Weightbearing
- Non-weightbearing

24. When you prescribe customised FOs for people with established rheumatoid arthritis, what manufacturing techniques are most frequently used to manufacture the FOs? (please choose one)

More info

- Computer aided manufacture (e.g. additive manufacturing such as 3D printing, or direct milling)
- Traditional manufacturing techniques (e.g. vacuum forming)

25. When you prescribe customised insoles for people with established rheumatoid arthritis what type of shell material do you most frequently specify? (please choose one)

More info

- Highly rigid (e.g. carbon fibre)
- Semi rigid (e.g. polypropylene)
- Semi flexible (e.g. high density EVA)
- Highly flexible / cushioning (e.g. medium or low density EVA)

26. When you prescribe customised insoles for people with established rheumatoid arthritis, what rearfoot posting do you most frequently specify? (please choose one)

More info

- None
- Intrinsic posting
- Highly rigid (e.g. acrylic / carbon fibre)
- Semi rigid (e.g. polypropylene)
- Semi flexible (e.g. high density EVA)
- Highly flexible/cushioning (e.g. medium or low density EVA)

27. When you prescribe customised insoles for established rheumatoid arthritis patients what top cover material do you most frequently specify? (please choose all that apply)

More info

- [ ] Minimal (e.g. leather / vinyl)
- [ ] Cushioning (e.g. Poron or similar polyurethane)
- [ ] Cushioning with specific modification to offload or cushion the forefoot
- [ ] Cushioning with specific modification to offload or cushion the midfoot
- [ ] Cushioning with specific modification to offload or cushion the rearfoot

# Thank you!

28. **Thank you for taking the time to complete this survey. If you would like to make any additional comments or provide us with further information, please do so below.**

29.

| | tick |
|---|---|
| If you would like a summary of the results sent to you, please tick this box and provide your email address below. | ○ |
| If you would like to enter the prize draw for the iPad mini, please tick this box and provide your email address in the space provided. | ○ |

29.a. Email (Please note that if you provide an email address, your response will no longer be completely anonymous, although we will not link specific answers to individual email addresses):

Please enter a valid email address.

## Thank you. You have completed the survey. There are no further questions. Please contact Robin Waxman, r.waxman@leeds.ac.uk, if you have any questions about this survey.

You can view, save and print this survey and your answers by clicking on **MY RESPONSES** above, and then **DOWNLOAD as PDF** on the following screen.

Australian podiatrists **MUST** print a copy of their responses to earn 1 hour of CPD.

---
