# Supplementary material for: A survey of foot orthoses prescription habits amongst podiatrists in the UK, Australia and New Zealand
Source: J Foot Ankle Res. 2018 Nov 26;11:64. doi: 10.1186/s13047-018-0304-z (PMC6258496; doi:10.1186/s13047-018-0304-z)
Supplement: Supplementary file 2 — Data from countries outside of the UK, Australia and New Zealand. (DOCX 16 kb) [file 13047_2018_304_MOESM2_ESM.docx]

**Supplementary data (other countries)**

Table 4 displays the countries of practice of the 29 respondents practising outside of the UK, Australia and New Zealand. Of these respondents, 18 (62%) were male. Respondents qualified between 1976 and 2016, with a mean (SD) of 15.6 (13.3) years since qualification. Five (17%) respondents worked solely in the public sector, 17 (59%) worked solely in the public sector, and 7 (24%) worked across both sectors. The two most frequently treated patient groups were non-inflammatory musculoskeletal and general practice (Table 5). Respondents prescribed more customised FOs per week than other FO types, and the majority had free choice when prescribing FOs (Table 6). Table 8 illustrates the prescription habits for different presentations and conditions.

**Table 4** Overview of the country of practice for those respondents who did not practice in the UK, Australia or New Zealand

| **Country of practice** | **No. of respondents** |
| --- | --- |
| Republic of Ireland | 10 |
| Canada | 4 |
| South Africa | 3 |
| United States | 2 |
| Singapore | 2 |
| Hong Kong | 1 |
| Italy/France/Spain | 3 |
| Channel Islands | 1 |
| Israel | 1 |
| Barbados | 1 |
| Romania | 1 |

*Note that one podiatrist selected both Republic of Ireland and Northern Ireland as their country of practice and was analysed as Republic of Ireland.*

**Table 5** Most frequently treated patient groups among respondents from other countries

| **Patient group** | **No. of respondents (%)** |
| --- | --- |
| Non-inflammatory musculoskeletal | 19 (65%) |
| General practice | 18 (62%) |
| Diabetes | 8 (28%) |
| Other (sports podiatry) | 3 (10%) |
| Paediatrics | 1 (3%) |
| Neurology | 0 (0%) |
| Systemic inflammatory diseases | 0 (0%) |

**Table 6** FO prescription habits among respondents from other countries

|  | **Simple FOs** | **PFFOs** | **CFFOs** |
| --- | --- | --- | --- |
| Mean (SD) (per week)  Range (per week) | 1.24 (3.8) 0-20 | 3.31 (6.9)  0-30 | 9.76 (20.2)  0-100 |
| No. (%) of respondents not prescribing FO type | 8 (28%) | 6 (21%) | 2 (7%) |
| No. (%) of respondents who had free choice when prescribing | 19 (90%) | 18 (78%) | 26 (96%) |

*PFFOs prefabricated functional foot orthoses, CFFOs customised functional foot orthoses.*

**Table 7** FO prescription habits for specific presentations and conditions among respondents from other countries

|  | **Not treated** | **No FOs** | **Simple FOs** | **PFFOs** | **CFFOs** |
| --- | --- | --- | --- | --- | --- |
| 1. **Back pain** | 5 (17.2%) | 5 (17.2%) | 1 (3.4%) | 6 (20.7%) | 12 (41.4%) |
| 1. **Hip pain** | 5 (17.2%) | 5 (17.2%) | 2 (6.9%) | 4 (13.8%) | 13 (44.8%) |
| 1. **Knee pain** | 1 (3.4%) | 3 (10.3%) | 0 (0%) | 9 (31%) | 16 (55%) |
| 1. **Patellofemoral pain** | 1 (3.4%) | 3 (10.3%) | 0 (0%) | 9 (31%) | 16 (55%) |
| 1. **Shin splints** | 1 (3.4%) | 3 (10.3%) | 0 (0%) | 10 (34.5%) | 15 (51.7%) |
| 1. **Ankle pain** | 1 (3.4%) | 5 (17.2%) | 0 (0%) | 7 (24.1%) | 16 (55.2%) |
| 1. **Achilles tendonitis** | 1 (3.4%) | 4 (13.8%) | 2 (6.9%) | 12 (41.4%) | 10 (34.5%) |
| 1. **Rearfoot pain** | 1 (3.4%) | 3 (10.3%) | 3 (10.3%) | 6 (20.7%) | 16 (55.2%) |
| 1. **Plantar heel pain / plantar fasciitis** | 1 (3.4%) | 3 (10.3%) | 1 (3.4%) | 9 (31%) | 15 (51.7%) |
| 1. **Peroneal tendonitis** | 1 (3.4%) | 4 (13.8%) | 1 (3.4%) | 9 (31%) | 14 (48.3%) |
| 1. **Tibialis posterior tendon dysfunction** | 1 (3.4%) | 3 (10.3%) | 0 (0%) | 4 (13.8%) | 21 (72.4%) |
| 1. **Midfoot pain/OA** | 1 (3.4%) | 3 (10.3%) | 1 (3.4%) | 9 (31%) | 15 (51.7%) |
| 1. **Forefoot pain/meta-tarsalgia** | 0 (0%) | 3 (10.3%) | 7 (24.1%) | 9 (31%) | 10 (34.5%) |
| 1. **Morton’s neuroma** | 1 (3.4%) | 4 (13.8%) | 7 (24.1%) | 9 (31%) | 8 (27.6%) |
| 1. **1^st^ MTPJ OA** | 1 (3.4%) | 3 (10.3%) | 3 (10.3%) | 7 (24.1%) | 15 (51.7%) |
| 1. **Diabetes without peripheral neuropathy** | 3 (10.3%) | 9 (31%) | 2 (6.9%) | 7 (24.1%) | 8 (27.6%) |
| 1. **Diabetes with peripheral neuropathy** | 4 (13.8%) | 4 (13.8%) | 3 (10.3%) | 4 (13.8%) | 14 (48.3%) |
| 1. **Non-inflammatory musculoskeletal disease** | 1 (3.4%) | 3 (10.3%) | 1 (3.4%) | 11 (37.9%) | 13 (44.8%) |
| 1. **Seronegative inflammatory arthritis** | 4 (13.8%) | 5 (17.2%) | 7 (24.1%) | 0 (0%) | 13 (44.8%) |
| 1. **Gout** | 5 (17.2%) | 12 (41.4%) | 2 (6.9%) | 6 (20.7%) | 4 (13.8%) |
| 1. **Connective tissue disease** | 9 (31%) | 7 (24.1%) | 0 (0%) | 5 (17.2%) | 8 (27.6%) |
| 1. **Neurological diseases** | 8 (27.6%) | 7 (24.1%) | 0 (0%) | 4 (13.8%) | 10 (34.5%) |
| 1. **Neuromuscular conditions** | 6 (20.7%) | 7 (24.1%) | 0 (0%) | 5 (17.2%) | 11 (37.9%) |
| 1. **Falls prevention in older adults** | 6 (20.7%) | 9 (31%) | 0 (0%) | 5 (17.2%) | 9 (31%) |

*PFFOs prefabricated functional foot orthoses, CFFOs customised functional foot orthoses, MSK musculoskeletal.*
